# A Linear-Quadratic Model for the Quantification of a Mixture of Two Diluted Gases with a Single Metal Oxide Sensor

**DOI:** 10.3390/s18061785

**Published:** 2018-06-01

**Authors:** Stéphanie Madrolle, Pierre Grangeat, Christian Jutten

**Affiliations:** 1 Univ. Grenoble Alpes, CEA, LETI, MINATEC Campus, Micro-technologies for Biology and Healthcare Division, F-38054 Grenoble, France; stephanie.madrolle@cea.fr; 2 GIPSA-lab, Univ. Grenoble Alpes, CNRS, Grenoble INP, F-38000 Grenoble, France; christian.jutten@gipsa-lab.grenoble-inp.fr

**Keywords:** metal-oxide, gas sensor, dual temperature mode, breath analysis, quantification, linear-quadratic model, acetone, ethanol, volatile organic compound (VOC)

## Abstract

The aim of our work is to quantify two gases (acetone and ethanol) diluted in an air buffer using only a single metal oxide (MOX) sensor. We took advantage of the low selectivity of the MOX sensor, exploiting a dual-temperature mode. Working at two temperatures of the MOX sensitive layer allowed us to obtain diversity in the measures. Two virtual sensors were created to characterize our gas mixture. We presented a linear-quadratic mixture sensing model which was closer to the experimental data. To validate this model and the experimental protocol, we inverted the system of quadratic equations to quantify a mixture of the two gases. The linear-quadratic model was compared to the bilinear model proposed in the literature. We presented an experimental evaluation on mixtures made of a few ppm of acetone and ethanol, and we obtained a precision close to the ppm. This is an important step towards medical applications, particularly in terms of diabetes, to deliver a non-invasive measure with a low-cost device.

## 1. Introduction

Gas sensors, especially metal oxide (MOX) gas sensors, are increasingly used in electronic noses to detect volatile organic compounds (VOCs) [1,2]. This is of particular relevance to medical applications because VOCs are abundant in breath [3,4]; several VOCs are biomarkers of disease [5,6,7] because their concentrations are different for healthy and ill persons. In the human body, acetone is generated by the liver according to a decarboxylation reaction of acetoacetate; most acetone exchanges take place in the airways, so acetone is present in exhaled air [8].Thus, analyzing breath is a non-invasive method for diagnosis or treatment follow-up. For example, acetone is linked to both diabetes [5,8] and weight loss [1]. Indeed, diabetics usually have an acetone rate between 2 and 7 ppm [8,9,10], sometimes reaching 21 ppm [11] whereas healthy people have less than 1 ppm in their breath [4,6,12]. Thus, estimating the concentration of acetone without blood sampling but with a portable breath analyzer would be a great advance for diabetics.

The MOX sensor is low cost, small, and reversible [13]; it presents all the necessary features for a portable device. However, because of its low selectivity, it is sensitive to several VOCs. In this work, we quantify a mixture of gases to detect the different compounds present in the mixture and estimate their concentrations. For such a goal, diversity, which cannot be obtained by a unique sensor, is required. Usually, sensing diversity is obtained by a multi-sensor system [1,14,15,16]. In other studies, previous authors have shown that modulating the temperature of a MOX sensor [17,18,19] changes the response of the sensor. Temperature modulation improves the sensitivity, helps to be discriminant [20,21], and can also improve the response time of a sensor [22]. Thus, changing the sensor temperature has a particular relevance in this work and, until recently, has been mainly used for gas detection and classification [14,16].

Our objective was to design a signal processing approach able to accurately quantify the main components of an exhaled breath gas mixture. The model we propose is based on signal-processing methodologies linked to inverse problems and source separation. Independent Component Analysis (ICA) is an example of such a method in which the measurements are modeled as a mixture of the gas components. In the simplest cases, this relationship (the mixture) is assumed to be linear. However, for a MOX sensor, a linear model does not hold. In this paper, we propose to model measurements as a linear quadratic mapping of the component concentrations. We will demonstrate that this model provides the best fit with our experimental data compared to other models proposed in the literature, improving the quantification accuracy.

For the evaluation on synthetic and experimental data, we considered, as proof of concept, mixtures of acetone and ethanol diluted in air. Acetone is one of the main VOC component of exhaled breath air and ethanol is one of its main interfering components. For a diabetic person who is willing to control their acetone breath content, which is linked to the glucose absorbed during their meal, we need to cancel the potential contribution of alcoholic beverages on the MOX signal. Furthermore, on real breath samples, which are far more complex, the objective will be to decompose the gas content into a concentration vector of elementary gas components. This vector would deliver the same measurements as the experimental one, based on the model we have established. A smaller subspace will be used to represent the data belonging to a much larger space. According to such a methodology, the acetone—ethanol dual gases mixture model presented here could be extended to represent more complex mixtures. But such a representation strongly relies on the acquisition model that will be used.

In this work, we propose to expand upon this theory and to quantify gas mixtures. First, we show it is possible to create virtual sensors by varying the temperature of the sensitive part of a unique MOX sensor. Moreover, by modeling the input/output relationships of the MOX sensor when the input is a gas mixture, we show that the sensing diversity obtained by varying the sensor temperature allows us to accurately quantify gas concentrations, even low concentrations. The paper is organized as follows. In Section 2, we describe the experimental device, the MOX sensor utilized, and the experimental conditions. In Section 3, the mixture sensing model is introduced. Compared to the literature, a new nonlinear model is proposed, based on the experimental data. In Section 4, we show that model inversion is achievable and accurately quantifies the sources, i.e., gas concentrations. In Section 5, our conclusion summarizes the main results and possible future works.

## 2. Experimental Set-Up and Method 

The aim of this work is to quantify a mixture of two gases diluted in an air buffer (79% of nitrogen and 21% of oxygen), with only a single sensor. Acetone is one of the VOCs with the highest concentration in exhaled breath air [23]. It is also linked to several clinical applications, such as diabetes or weight loss monitoring. Thus, we will consider acetone as the target gas. Ethanol is one of the main interfering gases to measure acetone with a MOX sensor. MOX sensors have been used for years for analyzing alcohol levels in breath. In such applications, it is known that acetone might interfere in the evaluation of alcohol concentration [24]. Thus, a mixture of acetone and ethanol diluted in air is a relevant model of exhaled breath air to test the analysis of the most abundant VOCs [23]. This ethanol interfering gas would simulate all the others gases present in breath. Also, as a diabetic person should evaluate their glucose uptake after a meal, the contribution of glucose uptake to acetone vapor production as well as the contribution of alcoholic drinks to ethanol vapor in the breath must be separated. Humidity is also a main breath component which interacts with MOX sensors. However, we assume that a hydrophobic filter (as proposed by CO2Meter [25], Intersurgical [26] or Perma Pure [27] for example) has been used to suppress a large part of this component before proceeding with the analysis. If the filter reduces the acetone or ethanol concentrations, it should mainly affect the coefficients but not the expression of the model. Therefore, an appropriate calibration should correct for the filter attenuation. Otherwise, a humidity correction can also be foreseen [28]. Humidity might be construed as an additional source; accordingly, adding a source term should compensate for the different values of humidity. This is not the aim of the actual study but this offers potential for future work.

To reach this goal, we acquired data with synthetic mixtures. The acquisition was divided into four steps. The first step was the preparation of the mixture. Thanks to the dilution of calibrated gas mixtures (produced by supplier), we obtained different samples. Then, we injected the mixture in an analytic cell of 200 mL in which the sensors were disposed. The gas flowed into the cell with a continuous flow rate of 500 mL/min. An exhalation contains between 500 mL and 2 L of air, as in the case of forced exhalation. VOCs are present at the end of the exhalation [29]. Accordingly, only the end tidal part requires analysis. If necessary, several end tidal exhalations could be analyzed. In a practical device, the gas would be stored in a tube [30] and a flowmeter would inject the gas with a constant flowrate. At the output, a voltage allowed us to find the resistance of the sensor linked to the redox reaction. The last step was the cleaning of the cell to prepare the device for another measure. This step must not be neglected because it contributes to the reproducibility.

With regard to the gas mixtures, we prepared 39 mixtures with different concentrations of acetone and ethanol. Acetone concentration varied between 0 ppm and 20 ppm, and ethanol one between 0 ppm and 40 ppm (corresponding to the typical breath content after the absorption of a small glass of wine, approximately 8 cl). To control the concentrations in the mixtures, flowmeters were used to dilute the calibrated mixtures, which were prepared by Air Products. This allowed us to obtain mixtures at the concentration we wanted to study. The performance of these flowmeters had been verified using a gas chromatography device using a flame-ionization detector (GC/FID) for the same range of acetone and ethanol concentrations as the one we used for this experiment. These concentrations were suited to our applications. Detection was achieved using a tin metal-oxide (MOX) sensor (SB-30 of FIS, FIS, Itami, Japan). According to the specifications given by FIS, this sensor is sensitive to a few ppm of ethanol and, according to Toyooka et al. [1], it is also very sensitive to acetone. Accordingly, it appeared well adapted to our objective of acetone and ethanol detection. Moreover, we noted that the sensor reacted when there was only 1 ppm of acetone in the synthetic air mixture. At a fixed temperature, the response time was relatively quick, around 30 s. The detection of the MOX sensor is based on an oxidation reaction. Indeed, in clean air, ions of oxygen are placed on the surface of a sensitive layer (tin oxide). When the sensor is exposed to a reducing gas (in this case, for acetone and ethanol), the oxygen reacts with the target gas. Thus, an oxidation reaction occurs between oxygen and reducing gas, releasing electrons and modifying the current [31]. As the voltage applied is fixed (5 V), the resistance of sensitive layer changes. Accordingly, the measure of resistance is directly linked to the composition of gas mixtures. The power dissipation of the sensing element is less than 10 mW.

For reproducible and relevant experimental data, we controlled and kept environmental parameters fixed because the gas sensor was sensitive to environment. Recordings were done with the same level of gas humidity, gas temperature, and oxygen concentration for all mixtures. Relative humidity was 1.67% ± 4.0%, temperature was 30.75 °C ± 0.55 °C, and oxygen concentration was 20.5% ± 0.31%. These parameters were controlled in the preparation of mixtures and measured in the analytic cell thanks to a SHT75 sensor (Sensirion, Staefa, Switzerland) for humidity and temperature, and with a O2/M-100 sensor (Membrapor, Wallisellen, Switzerland) for the oxygen. In these mixtures, the humidity level was low in comparison to that found in breath. Therefore, we propose to add a filter between the subject and the sensor. This filter will stop humidity; accordingly, the gas we analyze will be dry, with a humidity close to 0, as in the experimental conditions. The humidity will require adjustment when we consider real breath; however, in this study, the low humidity was in agreement with the breath humidity order of magnitude after a filter.

To have enough diversity for quantification, we proposed to modify the temperature of the MOX sensitive layer, thanks to a heater located under the sensitive layer. In the SB-30 sensor, the heater is platinum. The gas sensitive layer is also sensitive to the variations of temperatures [18,32], so the detection of gas particles by the MOX sensor will be different for each gas and for each temperature.

**Figure 1:**
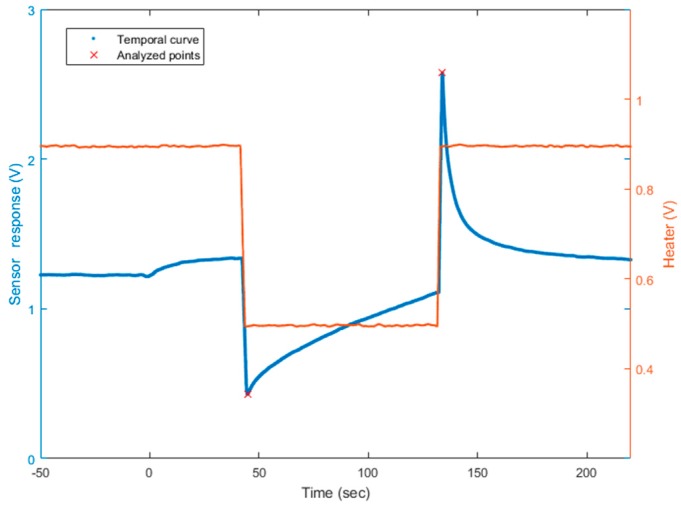
Sensor tension output according to the time (in blue, **left scale**) and the heater voltage applied (in red, **right scale**). The analyzed points are noted with a red cross on the temporal curve. The time t = 0 s corresponds to the injection of target gas in the analytic cell. Here, for example, the mixture is composed of 1 ppm of acetone and 4 ppm of ethanol, diluted in the air buffer.

**Figure 2:**
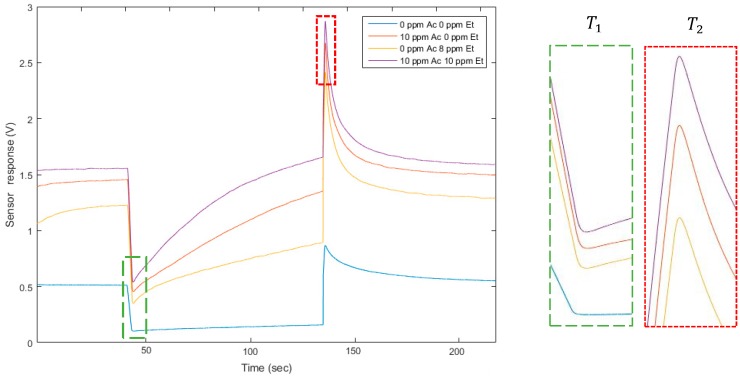
Sensor temporal responses presented for four mixtures of the two gases. On the right, enlarged portions on the analyzed points are presented for $T_{1}$ and $T_{2}$. The values of peak height for both temperatures are different according to the mixture composition.

Thus, to quantify two gases, we worked in a dual temperature mode. In this way, we obtained two virtual sensors with the diversity required for applying the source separation methods. To modify the temperature practically, we modified the voltage of the heater. We applied 0.5 V and 0.9 V. The website of the UST (Umwelt Sensor Technik) [33] provides the link between voltage applied on platinum heaters and the temperature of the heater on MOX sensors. Here, $T_{1}=$ 0.5 V corresponds to 131 °C, and $T_{2}=$ 0.9 V corresponds to 462 °C. The square signal applied to the heater lasted 90 s at each temperature, to allow the sensor to reach its steady state. The sampling frequency of the temporal curve was 500 Hz. On the raw curve, we applied a mean filter to cut the high frequency noise. Finally, we analyzed two points: the value of the peak height for both temperatures, after changing the heater temperature. One temporal curve was acquired in less than 4 min. Figure 1 illustrates the temporal curve, the heater voltage applied, and the points we analyzed on each curve. The presented methods and the presented model apply at any point on the temperature cycle. Choosing the peak height values was motivated by an optimization of the discrimination of the two gases [21]. They seem well appropriated and optimal to separate these two compounds. It also allowed a rapid delivery of a measurement immediately following temperature changes. The peak height includes the dynamic behavior of the sensor which is known to contain more information [17,22]. Additionally, the measures differed between acetone and ethanol. For a fixed concentration of acetone and an ethanol concentration varying (for example, 10 ppm of acetone, red and purple lines on Figure 2), the peak height was influenced by the ethanol concentration, varying here between 0 and 10 ppm. Similarly, for 0 ppm of ethanol (red and blue lines on Figure 2), the peak height changed when the acetone concentration was between 0 and 10 ppm. In addition to its rapid acquisition, the peak height varied according to the gas concentrations and, consequently, provided information about the mixture composition. 

Thus, for each sample of gas mixture, we acquired data in the air buffer alone ($V_{0}$) and in mixtures diluted in the air buffer ($V_{g}$). $V_{0}$, depending also on the temperature sensor, was also taken on the peaks heights. We calculated the resistance of sensitive using the equation given by the constructor [34]:

(1) $$R_{g}=R_{L}\left(\frac{4.55}{V_{g}}-1\right)$$

with $R_{L}$ a load resistance in the electronic editing. Data were acquired several times and we kept the mean values. The error bars were computed from several measurements, between 2 and 4 measurements, in the same configuration for a given temperature with the same sensor. At temperature $T_{i}$, we noted the measure $x_{i}$ (output of the MOX sensor), defined as the ratio of resistances (2):

(2) $$x_{i}={\frac{R_{0}}{R_{g}}|}_{T_{i}}$$

**Figure 3:**
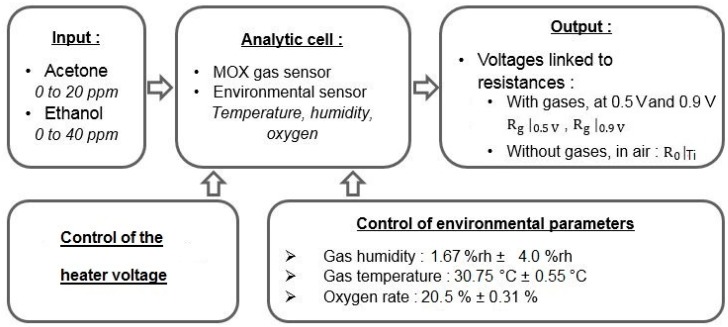
Schematic representation of the experimental device. We controlled two sets of parameters: (i) the environmental parameters, such as humidity and temperature and (ii) the sensor temperature, to obtain sensing diversity. At the sensor output, we measured the voltage from which we compute the resistances of the MOX sensor.

Figure 3 illustrates our experimental device.

## 3. Mixture Sensing Model

In this part, the parameters used to define the performance of the model are presented. Then, a step of model validation and a comparison with literature mixture models are shown. Finally, we calibrate the model by estimating the model parameters corresponding to the form previously defined.

### 3.1. Linear-Quadratic Model

In this section, we focus on the mixture model. It is known that, for a single gas, the resistance $R_{g}$ of a tin oxide MOX gas sensor follows the power law (3) [35]:

(3) $$R_{g}=A\cdot C_{g}^{r}$$

where *A* and *r* are coefficients to estimate and $C_{g}$ is the gas concentration. Concerning gas mixtures, we have found several, sometimes contradictory, models in the literature. Three models are shown in Table 1. For fixed temperature and humidity, Clifford [35] suggested a simple additive model (line 1 of Table 1), while Llobet [36] added a gas interaction term, and Hirobayashi [37] introduced a logarithmic model with an interaction term to achieve the best estimation. 

The aim of our study is to quantify a mixture of gases, thanks to the separation source methods. Currently, these methods use either linear [38] or bilinear models [39,40], or, for other applications like hyperspectral imaging, linear quadratic models [41], which are model candidates. However, these nonlinear models can be interpreted as a first-order Taylor approximation of a nonlinear function of mixtures of gas concentrations. Thus, we propose to refine the previous nonlinear model by considering a more accurate expansion with additional quadratic terms. We propose to consider a new nonlinear mixture sensing model, which is called linear-quadratic (line 4 in Table 1). We first introduced this model in Madrolle et al. [42], but only for modeling mixtures with high gas concentrations. Here, we propose that this model still holds for low concentrations in the range relevant for biomedical applications.

The linear-quadratic model consists of linear terms, bilinear (interaction), and quadratic terms of $c_{1}^{r_{1}}$ and $c_{2}^{r_{2}}$. In Section 5, we will discuss the relevance of adding these quadratics terms and the interest to keep all the second order terms, including quadratic terms, instead of only the bilinear interaction term.

As we used the MOX sensor at two temperatures, for each sample, we obtained two measurements, which led to the following system of two Equations (4). The exponent $r_{1}$ and $r_{2}$ are independent of the sensor temperature and so are the same in the two equations.

(4) $$\{\begin{array}{c} {{\frac{R_{0}}{R}}_{g}|}_{T_{1}}≃a_{11}C_{1}^{r_{1}}+a_{12}C_{2}^{r_{2}}+b_{11}{\left(C_{1}^{r_{1}}\right)}^{2}+b_{12}{\left(C_{2}^{r_{2}}\right)}^{2}+d_{1}C_{1}^{r_{1}}C_{2}^{r_{2}}+1\\ {{\frac{R_{0}}{R}}_{g}|}_{T_{2}}≃a_{21}C_{1}^{r_{1}}+a_{22}C_{2}^{r_{2}}+b_{21}{\left(C_{1}^{r_{1}}\right)}^{2}+b_{22}{\left(C_{2}^{r_{2}}\right)}^{2}\;+d_{2}C_{1}^{r_{1}}C_{2}^{r_{2}}+1 \end{array}$$

where $a_{ij}$, $b_{ij}$ and $d_{i}$ are characteristic of the temperature $T_{i}$ According to Llobet et al. [36], the exponent $r_{j}$ and the coefficients $a_{ij}$, $b_{ij}$, and $d_{i}$ are dependent on the couple gas/sensor. For a mixture without acetone and without ethanol, i.e., $C_{1}=0$ and $C_{2}=0$, the equations are consistent, since $R_{g}=R_{0}$ and so the ratio is equal to 1.

### 3.2. Model Performance Parameters

To estimate the performance of regression (Section 3.3) or of the inversion (Section 4.3), we used several parameters. First, correlation coefficient ρ (5) between the model ($x_{mod}$) and the measures ($x_{meas}$) and the relative error (6), in%, allowed us to qualify the model. We defined *i*∈[1…*N*] and *j*∈[1…*M*]. *N* and *M* are respectively the number of samples and the number of gases/temperatures and |u| and $\bar{u}$ denote the absolute and the mean values of u, respectively.

(5) $$\rho_{j}=\frac{1}{N-1}\overset{N}{\underset{i=1}{\sum }}\left(\frac{\bar{{x_{mod}}_{\iota ᴊ}-\mu_{x_{mod}}}}{\sigma_{x_{mod}}}\right)\left(\frac{{x_{meas}}_{ij}-\mu_{x_{meas}}}{\sigma_{x_{meas}}}\right)$$

(6) $$\sigma_{rel}{}_{j}\;\left(\%\right)=\frac{1}{N}\overset{N}{\underset{i=1}{\sum }}2\left|\frac{{x_{meas}}_{ij}-{x_{mod}}_{ij}}{{x_{meas}}_{ij}+{x_{mod}}_{ij}}\right|\cdot 100$$

Then, signal-to-noise ratio (SNR) (7) and mean square error (MSE) (8) will allow to examine the error on the source estimation.

(7) $${\mathrm{SNR}}_{j}=10\cdot {log}_{10}\left(\frac{\|s_{:j}{\|}_{2}}{\|s_{:j}-{\hat{s_{:ᴊ}}\|}_{2}}\right)$$

(8) $${\mathrm{MSE}}_{j}\left({\mathrm{ppm}}^{2r_{i}}\right)=\frac{1}{N}\overset{N}{\underset{i=1}{\sum }}{\left|\hat{s_{\iota ᴊ}}-s_{ij}\right|}^{2}$$

with $\hat{s_{\iota ᴊ}}$ as the estimated sources, $s_{ij}$ as the real sources for a sample *i* and a gas *j* and $s_{:j}$ as all the real sources *i* for a temperature *j*.

Thus, these parameters are used to characterize the performance of the model and the sources estimation.

### 3.3. Model Coefficients

**Figure 4:**
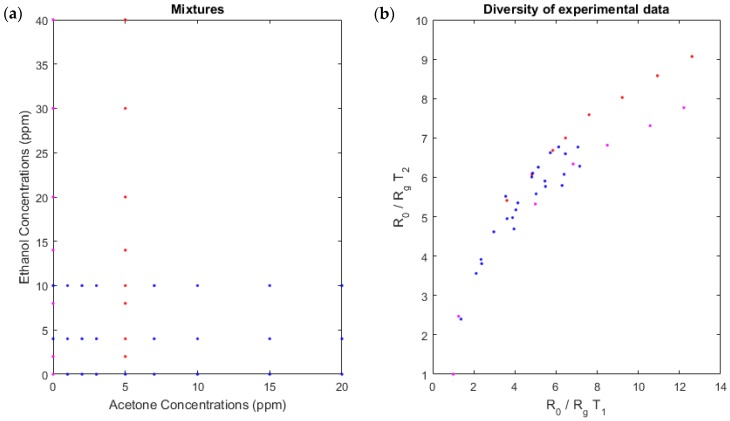
Diversity of experimental data used for calibration of our linear-quadratic model. (**a**) The concentrations of the two gases. These concentrations are known because we created a known mixture. (**b**) The diversity, the measures at temperature $T_{1}$ in function to measures at temperature $T_{2}$.

On the basis of this linear-quadratic formula of the mixture model, we needed to determine the coefficients $a_{ij}$, $b_{ij}$, and $d_{i}$. We calibrated our system with experimental data of known concentrations as detailed in Section 2. When we plotted the measures temperature $T_{1}$ in function to measures at temperature $T_{2}$ (Figure 4), the points were extended to a large area and the diversity obtained with experimental data appeared sufficient. Accordingly, the coefficients obtained by calibration were relevant.

We note that the diversity of measures for the peak heights at these two sensor temperatures is significant (Figure 4), which confirms the choice of the peak height points. The diversity will be discussed later (Section 4.1).

**Figure 5:**
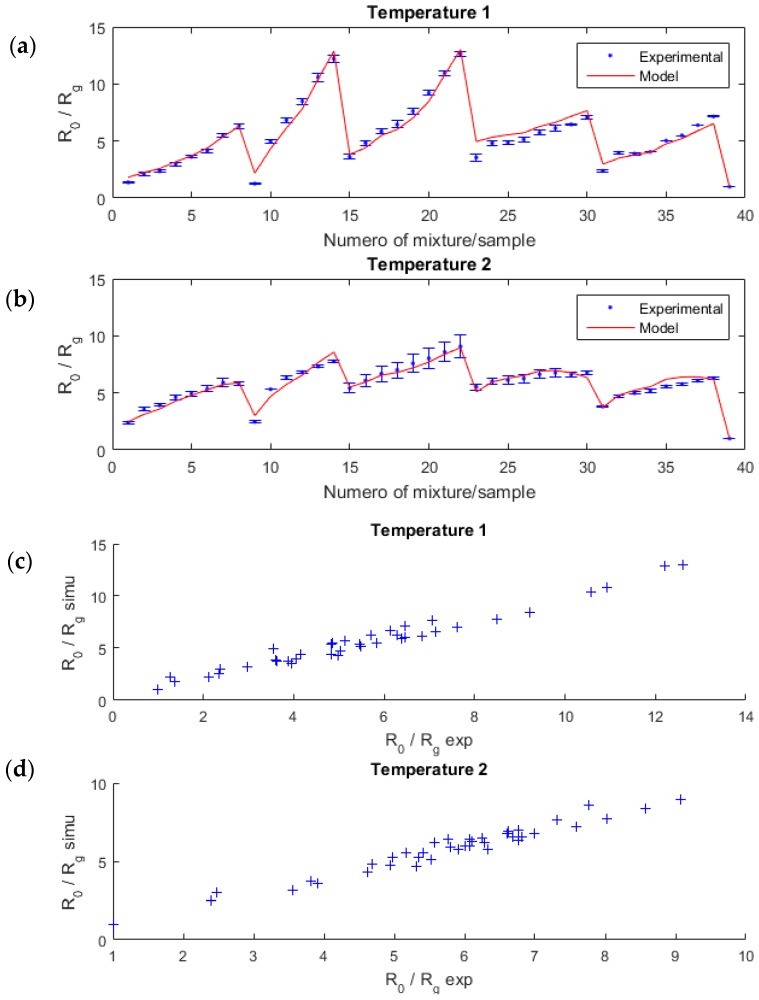
$R_{0}/R_{g}$ sensor resistance ratio for several mixture samples of acetone and ethanol (blue points), and the estimated linear-quadratic model (red lines) for each temperature (**a**,**b**). The error bars were computed from several measurements in the same configuration for a given temperature, with the same sensor. On (**c**,**d**) graphics, the correlation between the model and the experimental data is highlighted.

Thanks to a Levenberg-Marquardt algorithm, we estimated the 12 coefficients $a_{ij}$, $b_{ij}$, $d_{i}$ and $r_{j}$ (Table 2), which fit the best to the experimental data (Figure 5). The coefficients were different for each temperature, which illustrated the diversity, except for the exponent $r_{j}$, which we assumed to be the same for both temperatures to simplify the problem.

To evaluate the performance of our model, we computed the correlation coefficient ρ (2) between the model and the experimental data as well as the relative error (in%) (3). The model was in good agreement with the experimental data; the correlation was 0.97 and 0.96, respectively, for the first and the second temperature; the relative error was low, 6.7% and 3.6%, respectively.

To justify the relevance of the different terms (Table 3), we compared the performance of our model with another model from the literature. The linear-quadratic model achieved better performance, with a correlation of 0.96 and a relative error of 5.2%.

Thus, the regression performance parameters were the best with our linear-quadratic model. Even at low concentrations, this model described experimental data of a MOX sensor, which confirmed the relevance of the model, already observed at higher concentrations [42]. As the bilinear model appeared to have a performance close to our model, we kept it for evaluation and compare the results after inversion, as will be discussed in the next section.

The following depicts our linear-quadratic model in a compact form (9)

(9) $$x-1=As+Bs^{2}+ds_{1}s_{2}$$

where ***x*** is a vector, describing our observations for the two temperatures, $x={\left(x_{1},x_{2}\right)}^{T}$, ***s*** describes the sources, linked to the concentrations of the two gases, $s={\left(s_{1},s_{2}\right)}^{T}={\left(c_{1}^{r_{1}},\;c_{2}^{r_{2}}\right)}^{T}$ and $s^{2}={\left(s_{1}^{2},s_{2}^{2}\right)}^{T}$, ***A***, ***B*** are 2 × 2 matrices with general terms $a_{ij}$ and $b_{ij}$ respectively, and $d={\left(d_{1},d_{2}\right)}^{T}$ and $e={\left(e_{1},e_{2}\right)}^{T}$.

### 3.4. Cross-Validation

To validate the linear-quadratic model proposed, we selected a cross-validation methodology to estimate the reliability of the model. The method, also called “K-fold cross validation”, is based on a stochastic sampling technic. It consists of randomly dividing a set of samples into K clusters which have the same number of samples, forming a partition of the complete data set. K − 1 clusters are used for the learning (calibration) step. In our case, thanks to these clusters of known sources and the measures, we estimated the mixing coefficients with a Levenberg-Marquardt algorithm. The last sample subset was used for the validation step. Once we knew the model, thanks to the model coefficients found previously on the calibration samples, we verified the validation samples, using the known samples, that the simulated data were in agreement with the measure. These two steps were repeated until each sample had been used for the validation step. Finally, the mean square error (MSE) between the measures and the model output computed with estimated coefficients was calculated, to evaluate the performance of the model.

**Figure 6:**
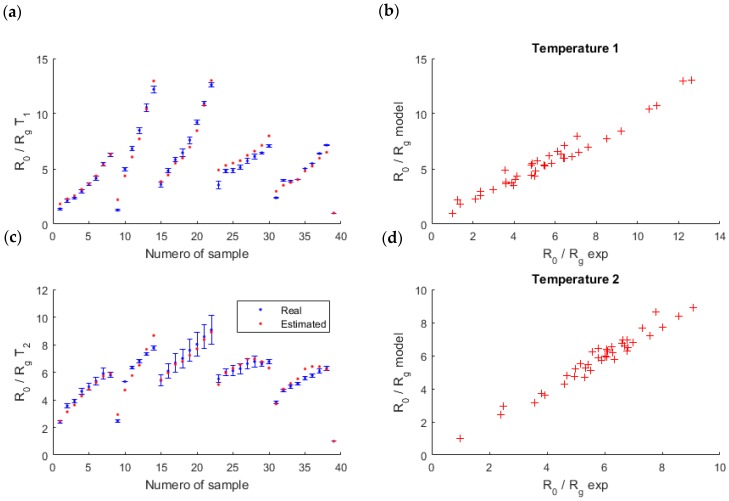
Validation of the model thanks to a 13-fold cross validation. On the left (**a**,**c**), the measures (in blue) and the mean of simulated data found during the validation steps of the 10 segmentations (in red). On the right (**b,d**), the link between the measures and the model. The results are shown for each temperature of the sensor ($T_{1}$ on the top and $T_{2}$ on the bottom).

We segmented the entire set of samples into K = 13 clusters of 3 samples, which corresponded to around 10% of the samples. In addition, to avoid a dependence of the segmentation chosen, we repeated this cross-validation 10 times with different segmentations, and we kept the mean of these 10 segmentations (Figure 6).

At the end, the mean of the 10 MSE obtained (for each segmentation) was low, $0.28\;\mathrm{pp}m^{{2r}_{i}}$, which validated the model: the linear-quadratic model fits well to the measures.

## 4. Model Inversion

We evaluated the performance on the source concentrations estimated by inversion of this model to confirm our choice of two sensor temperatures and to validate the linear-quadratic model for this application. We will first consider simulated data and then the experimental data.

### 4.1. Simulated Data

**Figure 7:**
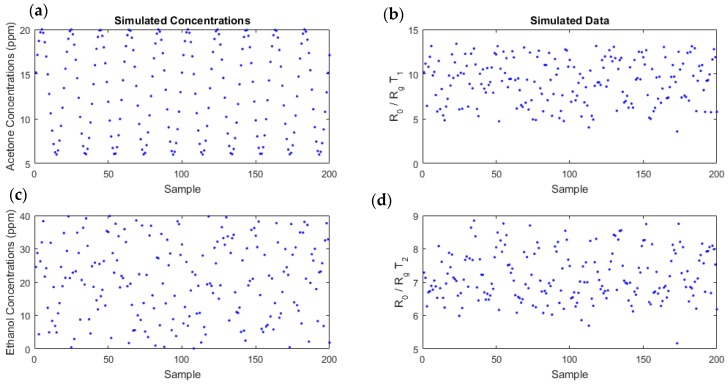
Simulated data (**b**,**d**), obtained using the linear-quadratic model, with an additive Gaussian noise for temperatures $T_{1}$ (**b**) and $T_{2}$ (**d**), for the concentrations (**a**,**c**). Acetone concentrations were sinusoidal time series varying between 0 and 20 ppm (**a**) and ethanol concentrations followed a uniform distribution, between 0 and 40 ppm (**c**).

Using the linear-quadratic model with the coefficients estimated from experimental data (Table 2), we generated realistic simulated data. We chose to vary the acetone concentrations according to a sinusoidal law, between 0 and 20 ppm. The sinusoidal law could correspond to the variation of acetone in diabetic individuals; for example, acetone can increase during the night or during physical effort. For ethanol, we randomly drew a sample with a uniform distribution, between 0 and 40 ppm. These concentrations series evolved differently with independent variations. A Gaussian noise, whose variance was equal to the MSE of the model, was added, which led to a signal-to-noise ratio (SNR) around 19 dB. *N* = 200 points were thereby simulated (Figure 7).

**Figure 8:**
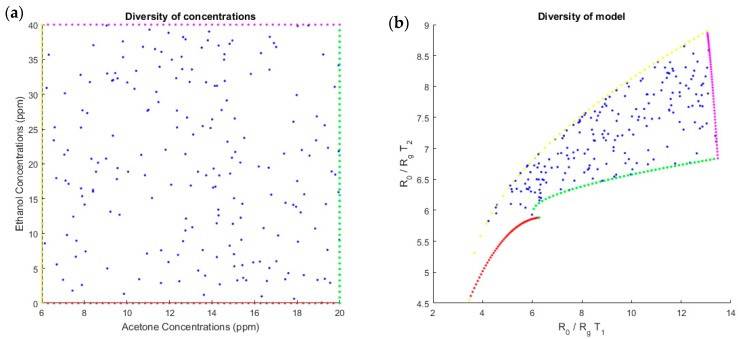
Scatter plot of the dual-temperature MOX sensor outputs, based on the linear-quadratic model (**b**) in response to the sampled concentrations of acetone and ethanol (**a**). The concentrations follow a uniform distribution to show the diversity of the entire model. Each point on the right diagram shows the link between the sensor output at temperature $T_{1}$ along the abscise axis, and the sensor output at temperature $T_{2}$ along the ordinate axis.

A necessary condition to correctly invert our system was to have enough diversity and a one-to-one input/output mapping in the concentration range. For one mixture, measures at each temperature could not be proportional (i.e., measures at temperature $T_{1}$ in function to measures at temperature $T_{2}$ could not belong to a one-dimensional manifold with a volume equal to 0). We wanted diversity in measures. To verify these points, we computed the response $x_{i}$ for each temperature obtained by sampling each concentration randomly with a uniform distribution on the working interval. The scatter plot of (uniformly distributed) input samples are represented in the source plane, i.e., $\left(s_{1},s_{2}\right)=\left(c_{1}^{r_{1}},\;c_{2}^{r_{2}}\right)$ (Figure 8a), and the mapping of these samples by the linear-quadratic model in the plane $\left(x_{1},x_{2}\right)$ is represented in Figure 8c. For acetone concentrations between 6 and 20 ppm and ethanol concentrations between 0 and 40 ppm, because the shape of the sampled region was not too flat and appeared to be without folding, we can conclude that the diversity was sufficient for this range of concentrations. We confirmed this by inverting the model. Similarly, for the simulated data (Figure 8b), the diversity in ratio of resistances for the two temperatures was good because the measures at two temperatures were not proportional.

**Figure 9:**
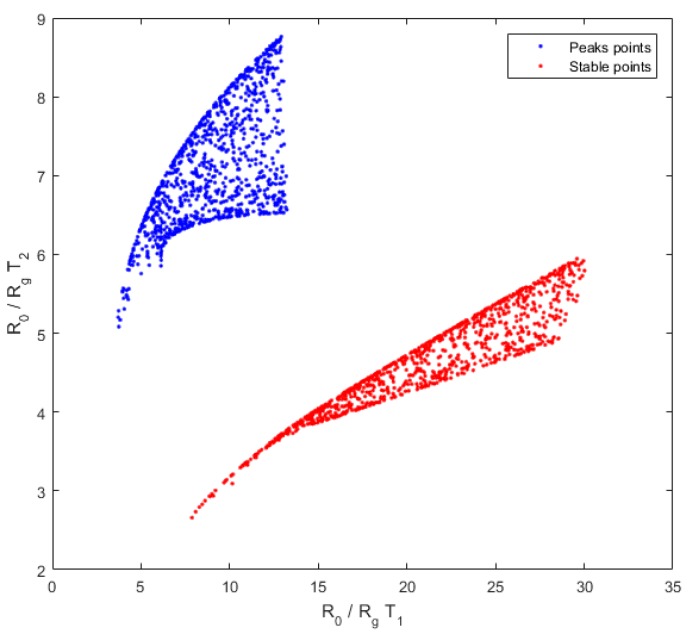
Diversity of stable points (red) and peak height points (blue), the coordinates of each measurement point are defined by the resistance ratio at $T_{1}$ in abscissa and at $T_{2}$ in ordinate. A great diversity is characterized by a scattered graph. Here, the peak height points seem more dispersed. Accordingly, the diversity for the peak height points is better and the discrimination between gases will be better for this time point of the temporal curve.

The diversity of measures, which is linked to the discrimination power of gases, is better for the peak height points than for the stable points (Figure 9). Indeed, the graph for the stable point is elongated, whereas the graph for the peak height points is more dispersed. This confirms that using peak height points will have a better discrimination power than using the points after output voltage stabilization.

### 4.2. Inversion Method

In this section, we inverted our model, i.e., to estimate the concentrations $\left(c_{1}^{r_{1}},\;c_{2}^{r_{2}}\right)\;$ using the measures (or the simulated data) ***x***, the formula of the mixing model (9), and the coefficients ***A***, ***B*** and ***d***. We looked for the sources ***s*** which allowed us to find the concentrations of our mixtures. The method of Levenberg-Marquardt [44], which is a least squares method, was used to estimate the sources. In this method, the cost function is the following (10).

(10) $$f_{\mathrm{cost}}\left(s\right)=\overset{N}{\underset{i}{\sum }}\|As_{i}+Bs_{i}^{2}+ds_{i1}s_{i2}+1-x_{i}\|{}^{2}$$

where *N* is the number of sample (corresponding to each mixture).

Thus, we minimized this cost function to find the best source vector ***s***, corresponding to the measurement vector ***x***.

To minimize this cost function, the Levenberg-Marquardt method solve, for each iteration *k*, the normal Equation (10), with the unknown direction $d_{k}$ defined by the following equation:

(11) $$(J\left(s_{k}\right){\;}^{T}J\left(s_{k}\right)\;+\lambda_{k}\;I\;)\;d_{k}=J\left(s_{k}\right){\;}^{T\;}f_{cost}\left(s_{k}\right)$$

where $J\left(s_{k}\right)$ is the jacobian of $f_{cost}$ in $s_{k}$ and $\lambda_{k}$ is a regularization parameter, which tends toward 0 when the solution approaches. Subsequently, the solution ***s*** is updated (12):

(12) $$s_{k+1}=s_{k}+d_{k}$$

This iterative method requires the correct initialization of the sources. If the initial sources $s_{0}$ are too far from the real sources, the algorithm may converge toward a local minimum, which is not the minimum we looked for. When we worked with the simulated data, we knew the range in which the sources were included, and we initialized the recurrence series (12) at the middle of this range. With experimental data, we suppose we approximately knew the sources, with an error of 20%.

### 4.3. Results

We present the results for the simulated and experimental data.

#### 4.3.1. Simulated Data

**Figure 10:**
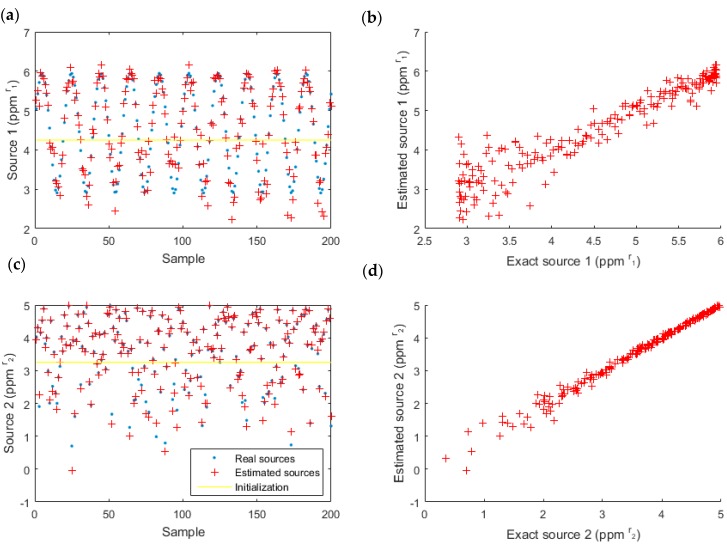
Estimated data for source one (acetone) (**a**,**b**) and source two (ethanol) (**c**,**d**), with the linear-quadratic model. The real sources are also represented (**left**) to compare the estimation and the reality and the correlation is highlighted (**right**).

First, we computed the simulated data using a linear-quadratic model and coefficients which had been experimentally estimated (Table 2). Then, we computed the sources using an inversion of the same model (Figure 10 and Table 4) to check if the model was invertible and to test its robustness. 

The estimated sources and the actual sources (simulated) are almost superposed (left), and the difference is low (right) for the two sources. The performance parameters (Table 4) confirm these visual conclusions because the SNR was 12.9 dB and the mean square error (MSE) was 0.07 pp$m^{2r_{j}}$.

#### 4.3.2. Experimental Data

**Figure 11:**
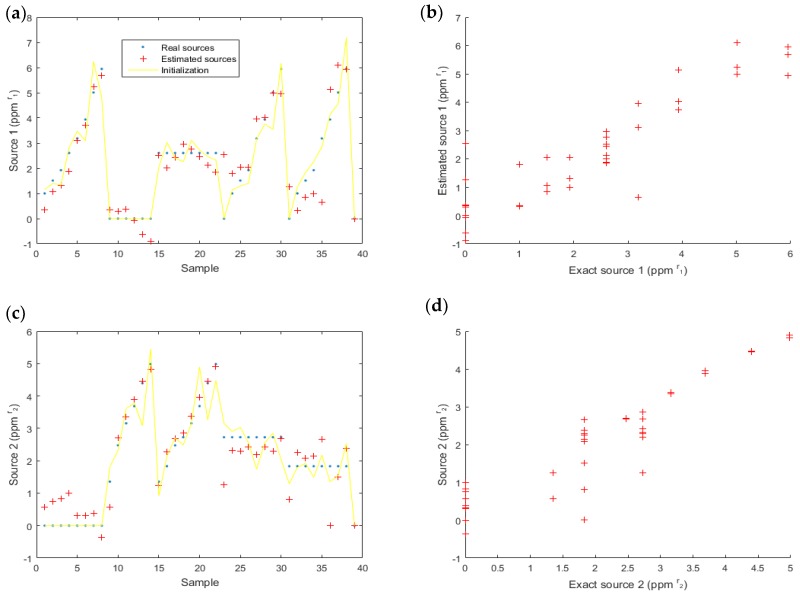
Estimated data for source one (acetone) (**a**,**b**) and source two (ethanol) (**c**,**d**). The real sources are also represented to compare the estimated and the actual values (**a**,**c**) and the correlation between real and estimated source is highlighted (**b**,**d**). On (**b**,**d**), each color corresponds to one value of the other source.

Next, we considered experimental data (Figure 4). On these data, the results (Figure 11) were satisfactory. For the first source (acetone), the SNR = 5.6 dB and MSE = 0.67 pp$m^{2r_{1}}$; for the second source (ethanol), SNR = 6.4 dB and MSE = 0.34 pp$m^{2r_{2}}$.

## 5. Discussion

The results show a good estimation of the sources, particularly on simulated data, with high correlation, SNR, and a low MSE. This proves that the nonlinear system (4) associated with the two-temperature virtual sensors is well-conditioned.

With regard to the simulated data, if we transform the sources in concentrations, thanks to a change in variables, we may estimate the concentrations of two gases with a precision inferior to 1 ppm. Working with low concentrations and obtaining such a precision allows us to be closer to the targeted medical application. 

With regard to the experimental data, after a conversion of sources in concentrations, we achieved a precision on concentrations less than 1.5 ppm for both gases (acetone and ethanol). The results are encouraging. This precision of 1.5 ppm for acetone could allow detection for all types diabetes (Type 1 and Type 2). For diabetics with higher acetone breath, the error of detection would be lower and the diagnosis would be more reliable, in comparison to diabetics with a lower acetone rate. In contrast, for healthy subjects, the precision required is less than 1 ppm; for this purpose, we can consider a person healthy if he/she is not diabetic. The limited number of measures may explain the error. With more than 39 experimental points, estimation would improve because the criterion of independence of sources would be more accurate. To improve the quality of estimation, one could to add a priori information about, for example, the range of the sources or of the mixing coefficients [45]. A method including this information could improve the results.

Next, we compared the two better models selected in Section 3: the linear-quadratic model (our model), whose results are presented in Section 4, and the bilinear model. To obtain the results of bilinear model, we found the coefficients associated with the model and we inverted the problem with the same method (Section 4.2) using experimental data. Therefore, we can compare the results of these two models (Table 5).

The performance parameters were better for the linear-quadratic model than for bilinear model in term of correlation, of SNR, and of error. This implies that the choice of model impacts the estimation of sources; choosing a good model allows for higher precision in our final goal to estimate the concentrations.

## 6. Conclusions

In this paper, we present a complete work, including acquisition of experimental data, design, and validation of the sensing model, as well as inversion of the model for recovering the gas concentrations. The results allow us to validate several points. First, we validated the dual-temperature mode, which enabled the diversity required to quantify a mixture of two gases. This mode provided two virtual sensors with sufficient diversity, using a unique physical MOX sensor. Then, we proposed and validated a linear-quadratic model for modeling the MOX sensor mapping in response to a mixture of gases. In comparison to the models described in the literature, the regression was improved by adding quadratic terms. It also impacted the final estimation of the concentrations. Moreover, we validated the inversion by estimating the sources based on the measures (i.e., MOX sensor outputs). In comparison to the bilinear mixture model, our model achieved a better SNR. These three points have been validated for low concentrations, between 0 and 20 ppm for acetone and 0 and 40 ppm for ethanol. These results are consistent with the targeted medical applications, and this paper presents all the steps necessary to quantify a mixture of two gases in an air buffer. 

Thus, the advantages of this new model are to compute data output which fit the best with experimental measurements. In addition, combined with a method of source separation for studying gas mixtures, the quantification of the concentration of each gas component increased in accuracy. Indeed, the estimation method provided better results with a nonlinear model, close to reality.

The next step was to avoid calibration. Model coefficients were estimated using experimental data with different mixtures, which required a long time and a high degree of accuracy. Instead of this supervised approach, future work will consider approaches based on blind source separation [46,47,48] for estimating the coefficients of the model without knowing exactly the concentrations of the mixtures. This will extend the preliminary results presented in Madrolle et al. [42,49] to other source separation methods and to sources with low concentrations. In addition, we will increase the complexity of the mixture by adding gases, to be closer to the complexity of human breath. The goal is to apply the proposed method directly to human breath. Also, humidity, naturally present in human breath, might be taken into account as an additional source, defined by a non-integer power of the gas concentration and included in a second order polynomial model. For complex samples, thanks to the model, we will be able to reduce the sample complexity to a smaller sub-space. We will compute the concentration vector of a mixture of acetone and ethanol which would provide the same measurements as the experimental ones based on the relationship defined by the model. Such a fingerprint will help to discriminate between samples. The model can be upgraded by adding additional source terms to the linear quadratic expression. However, in such cases, complementary measurements will be required for source quantification. 

## Figures and Tables

**Table 1:** Comparison of mixture model formulas in literature for an ${\mathrm{SnO}}_{2}$ MOX sensor.

Model	Equation	Reference
Linear	$R_{0}/R_{g}-1=A_{1}C_{1}^{r_{1}}+A_{2}C_{2}^{r_{2}}$	[43]
Bilinear	$1/R_{g}-1/R_{0}=A_{1}C_{1}^{r_{1}}+A_{2}C_{2}^{r_{2}}+A_{3}C_{1}^{r_{1}}C_{2}^{r_{2}}$	[36]
Logarithmic	$R_{g}=A_{1}+A_{2}\log\left(C_{1}\right)+A_{3}\log\left(C_{2}\right)+A_{4}\log\left(C_{1}\right)\log\left(C_{2}\right)$	[37]
Linear-quadratic	$R_{0}/R_{g}=A_{1}C_{1}^{r_{1}}+A_{2}C_{2}^{r_{2}}+B_{1}C_{1}^{2r_{1}}+B_{2}C_{2}^{2r_{2}}+d_{1}C_{1}^{r_{1}}C_{2}^{r_{2}}+1$	[42]

**Table 2:** Coefficients of the system of Equation (3), estimated by least square regression.

	$a_{1i}$	$a_{2i}$	$b_{1i}$	$b_{2i}$	$d_{i}$	$r_{1}$	$r_{2}$
$T_{1}$	80	0.32	0.15	0.41	−0.16	0.60	0.44
$T_{2}$	61	1.47	−0.13	0.01	−0.22		

**Table 3:** Comparison of the performance parameters, correlation and relative error, for each model. We considered four models: the linear-quadratic model (Lin.-Quadr.), the linear model (Lin.), the bilinear model (Bilin.), and the logarithmic model (Log.).

	Lin.-Quadr.	Lin.	Bilin.	Log.
ρ	0.96	0.91	0.95	0.36
$\sigma_{rel}$ (%)	5.2	8.1	5.3	18

**Table 4:** Performance parameters: correlation and signal-to-noise ratio (SNR) between estimated and real values for each source. The model used is the linear-quadratic one.

Sim. Data	ρ	SNR
$s_{1}$	95	11.2
$s_{2}$	99	14.5

**Table 5:** Comparison of two models, linear-quadratic and bilinear, thanks to performance parameters, correlation, signal-to-noise ratio (SNR), and mean square error (MSE), between estimated and actual sources. The experimental data are used for this comparison.

Exp. Data	ρ	SNR	MSE (ppm^2r_j_^)
Linear-Quadratic	0.91	6	0.5
Bilinear	0.57	3.5	9
